# Food safety and irradiation: protecting the public from foodborne infections.

**DOI:** 10.3201/eid0707.017706

**Published:** 2001

**Authors:** R V Tauxe

**Affiliations:** Centers for Disease Control and Prevention, Atlanta, Georgia 30333, USA. Rtauxe@cdc.gov


### Food Safety and Irradiation: Protecting the Public from Foodborne Infections

**Robert V. Tauxe**
Centers for Disease Control and Prevention, Atlanta, Georgia, USA

Early in the 20th century, when food safety was a major concern to the public, two technologies, milk pasteurization and retort canning, were developed, promoted, and virtually canonized as prevention measures against foodborne diseases. Fear of contracting typhoid fever from watered milk and outbreaks of botulism from commercially canned products are now part of the distant past, controlled by these food industry processes in many countries. Nonetheless, at the beginning of the 21st century, foodborne disease remains a major threat to public health, as new pathogens and products have emerged (1). Many of these threats can be controlled by applying new technologies, when we as a society are willing to use them.

In the United States, foodborne infections cause an estimated 76 million cases of illness and 325,000 hospitalizations annually—more than 1 in 1,000 are hospitalized each year (2). The economic burden is substantial, estimated at up to $6.7 billion annually in patient-related costs for treatment of bacterial infections alone (3). Five pathogens account for much of the most severe illness: *Salmonella*, *Escherichia coli* O157 and other Shiga toxin-producing *E. coli*, *Campylobacter, Listeria*, and *Toxoplasma* cause an estimated 3.5 million infections, 33,000 hospitalizations, and 1,600 deaths each year (2).

In the early 1990s, large and devastating foodborne outbreaks of *E. coli* O157:H7 infections heightened public concern about foodborne diseases (4). Efforts to improve food safety were intensified in industry and regulatory agencies and supported by the National Food Safety Initiative (5). As a result of these efforts, the process control strategy of the Hazard Analysis-Critical Control Point (HACCP) is becoming the norm to use for producing many foods. In slaughter inspection it is replacing manual and visual carcass-by-carcass inspections. An expanded focus on regulating sanitation and hygiene with good manufacturing and agricultural practices means that food would be produced under cleaner conditions. In restaurants and home kitchens, new attempts have been made to educate food preparers in the basic principles of food safety, though paid sick leave for foodhandlers is still the exception, and handwashing is intermittent. These developments may collectively help explain a decline in the reported incidence of *Salmonella* and *Campylobacter* infections that was observed in active surveillance by FoodNet between 1996 and 2000 (6). However, we are still far from the public health goals established for 2010. These goals include reducing the national incidence of infections with *Salmonella*, *E. coli* O157, *Campylobacter*, and *Listeria* to 50% of their 1997 incidence (7). Reaching those goals means preventing 50% of foodborne diseases now occurring. This will require new approaches for prevention.

### Traditional Methods: Sanitation and Pasteurization

In general, effective vaccines are not available to protect against pathogens that cause foodborne diseases, either for immunizing humans or for animals that serve as hosts and may be eaten by humans. Educating foodhandlers, consumers, and food producers in basic food safety is important but is not sufficient by itself. Protecting consumers from the most severe diseases has been achieved by increasing the safety of food along the chain of production, from farm to table (Figure 1). For many foodborne infections, control has been most successful when mechanisms of transmission are understood well enough to prevent contamination from occurring before consumers purchase food. This has meant rethinking food production processes and sometimes introducing new safety steps to reduce levels of microbial contamination. The degree of safety built into the process varies, depending on the risk and the technologies available to address the risk.

For all foods, using basic principles of sanitation and food hygiene preserves wholesomeness and shelf life. For foods susceptible to contamination with particularly deadly

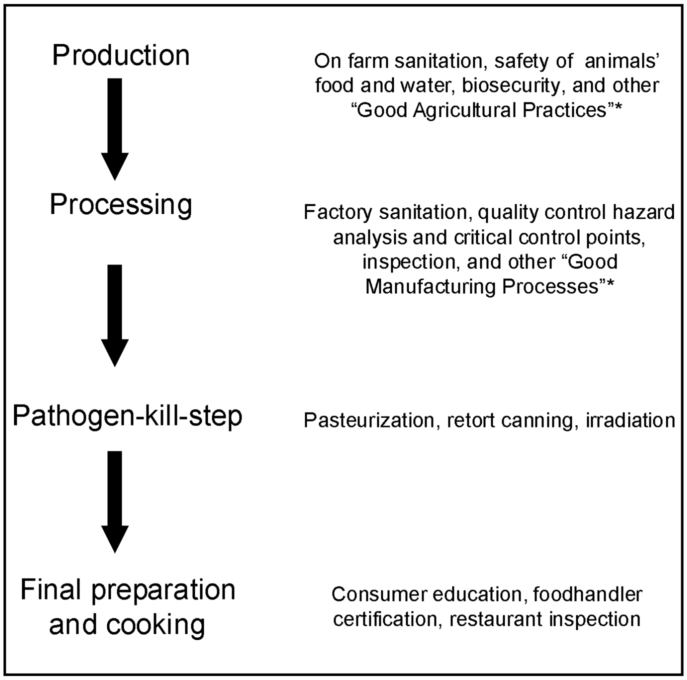

Figure. The chain of food production and foodborne disease prevention from farm to table. *These are terms used by FDA as guidelines for agriculture and food manufacturing practices.

Address for correspondence: Robert Tauxe, Centers for Disease Control and Prevention, 1600 Clifton Road, MS A38, Atlanta, GA 30333, USA; fax: 404-639-2205; RTauxe@cdc.gov pathogens, we as a society have demanded that additional protective measures be taken to eliminate those pathogens from the food altogether. A definitive food safety measure is necessary when contaminated food products put the general population at risk for severe illness and when typical food production practices do not eliminate the risk reliably, especially those pathogens that cause severe illness to humans exposed to even small amounts. It is not sufficient to rely on routine foodhandling practices in the kitchen to prevent illness if the pathogen is already present in the food. In the past, it has often been a catastrophe resulting from a large foodborne outbreak that spurs the demand for new measures to completely eliminate the pathogen from food.

Implementation of definitive new measures for food safety has historically been slow. For example, canning was widely practiced as a means of preserving food in the 19th century, but methods were not standardized. The principal risk associated with eating improperly canned foods is botulism, a devastating paralytic illness that follows ingestion of food containing botulinum toxin. Botulinum is an extremely potent toxin produced by the bacterium *Clostridium botulinum* under certain anaerobic conditions, such as those that may be found inside a hermetically sealed can. This bacterium can live inside a can because it forms a hardy spore that can survive the temperature at which water boils at ordinary air pressure. It takes temperatures higher than 100 degrees Celsius to kill spores in canned food.

Before the invention of artificial ventilation and intensive care, half of those who contracted botulism died, and even now, botulism means many weeks in intensive care. Large outbreaks during and following World War I drew attention to the public health hazard of poorly canned foods. A 1919 multistate outbreak that resulted in 15 deaths was traced to canned ripe olives from California (8,9). This outbreak led to the development in 1923 of an industry standard method for cooking food at high enough temperatures to kill the botulinum toxin, the so-called botulism retort cook. This method reliably reduced clostridial spore counts by 12 decimal logs, the highest conceivable level of contamination (10). In 1930, a federal standard for quality of canned foods was developed, because of concern that vegetables that were canned might be of inferior quality (11). However, it was not until 1973, following an outbreak of botulism traced to defectively canned commercial vichysoisse soup (12), that the current federal regulation of canned foods was passed.

Pasteurization of milk, another fundamental technology used to prevent foodborne disease, was also adopted slowly over many years. At the turn of the last century, cows' milk was recognized as the source of a large number of different infections, including typhoid fever, bovine tuberculosis, diphtheria, and severe streptococcal infections (13). A commercial pasteurizer was patented in Germany in 1893, and, by 1900, a standard set of pasteurization conditions were defined, based on the time and temperature required to inactivate *Mycobacterium tuberculosis*, which was thought to be the most heat-resistant pathogen. However, pasteurization was opposed because it was believed that it might be used to market dirtier milk and also because of fears that it might affect the nutritional value of milk (14); therefore, the technology was implemented slowly. For some, the best way to prevent infections spread through milk was to pay scrupulous attention to the health of animals and to create sanitary conditions for the milk production process. This "certification movement" led to substantial improvements in dairy conditions. However, recurrent outbreaks of illness traced to some certified dairies clearly indicated a need for pasteurization. Initially, different jurisdictions adopted either improved sanitation or pasteurization. The requirements of the Public Health Service Standard Milk Ordinance in 1927 combined the two strategies: first, milk was to be graded based on a variety of sanitation measures; second, only Grade A milk could be pasteurized (15). By the end of the 1940s pasteurization was heavily promoted throughout the industry and became the norm. Now, 99% of fresh milk consumed in the United States is pasteurized, Grade A (16).

The use of both retort canning and milk pasteurization took decades to gain universal acceptance. Many were concerned that the use of these technologies would lead to slippage of standards for quality and sanitation. These concerns were ultimately addressed by using formal grading processes to assure the public that only clean milk would be pasteurized, and only vegetables of clearly defined quality would be canned. Concerns that loss of nutrients would be an important issue were found to be unwarranted. Although a wide variety of times and temperatures were initially used, clear microbial target endpoints were ultimately defined for both canning and pasteurization so that milk pasteurization and botulism retort cook have standard meanings everywhere in the United States. Quality grading standards and pathogen elimination processes were first developed by the industry and then formally adopted via federal regulation. Both processes are generally applied to foods that are either packaged or that will be immediately packaged. This method minimizes the opportunity for posttreatment recontamination.

Use of these processes has eliminated outbreaks of botulism in commercially canned food and has made outbreaks of illness spread through milk extremely rare. Foodborne botulism is now a rare illness, with approximately 25 cases a year that are almost always associated with home-canning or home-preserving (16). Bovine tuberculosis, typhoid fever, and septic sore throat resulting from milk tainted with bacteria have disappeared. Outbreaks of infections due to unpasteurized milk still occur (16). The rare outbreaks that are traced to pasteurized milk are usually the result of breaks in postpasteurization hygiene (16,18).

### Today's Technology: Food Irradiation

The use of high energy irradiation to kill microbes in food was evaluated in this country as early as 1921, when scientists at the United States Department of Agriculture reported that it would effectively kill trichinae in pork (19). Irradiation has become a standard process used to sterilize many consumer and medical products, from adhesive strips to surgical implants. Three different technologies that can be used to treat food have been developed by the sterilization industry.

### Gamma Irradiation

Gamma irradiation technology uses high energy gamma rays that are emitted by radioactive cobalt 60 or cesium 137. These radioactive sources are produced in commercial nuclear reactors and have a long half-life that makes them useful for commercial installation. Food or other products are brought into a heavily-shielded chamber and exposed to gamma rays for a defined length of time. When the source is not in use, it is stored in a pool of water that absorbs all irradiation, effectively turning it off. These high energy rays can penetrate deeply, making it possible to treat bulk foods on shipping pallets.

#### Electron Beam Irradiation

Electron beam technology uses a stream of high energy electrons, also known as beta rays, that are emitted from an electron gun. The technology is analogous to an electron beam in a television tube, though far more powerful. Electrons can only penetrate several centimeters of food, and for this reason, foods are treated in relatively thin layers. Modest metal shielding of the treatment cell is sufficient to prevent the escape of stray electrons. When not in use, the electron source is turned off by switching off the electric current. No radioactivity is involved.

#### X-Irradiation

The most recently developed technology, X-irradiation, mixes properties of both of the above. High energy X-rays can be produced if an electron beam hits a thin metal foil target. Like gamma rays, a beam of X-rays can penetrate foods to a much greater depth than electron beams and requires heavier shielding. However, like electron beams, X-ray sources can be switched on and off and do not use a radioactive source.

### Effect of Irradiation on Microbes

The high energy rays of irradiation directly damage the DNA of living organisms, inducing cross-linkages and other changes that make an organism unable to grow or reproduce. When these rays interact with water molecules in an organism, they generate transient free radicals that can cause additional indirect damage to DNA. An absorbed dose of irradiation energy is now measured in units called Grays, rather than an older measure called a rad. One Gray equals 100 rads, and 10 kiloGray equal 1 megarad. Complex life forms with large DNA molecules are affected by relatively low doses. Simpler organisms with smaller DNA can take progressively higher doses. Thus, a low dose of under 0.1 kiloGray kills insects and parasites and inhibits plants from sprouting. A medium dose, between 1.5 and 4.5 kiloGray, kills most bacterial pathogens other than spores, and a higher dose of 10 to 45 kiloGray will inactivate bacterial spores and some viruses. Prions, which do not contain nucleic acid, are difficult to inactivate by irradiation. For humans, the lethal dose is 4 Gray.

The actual dose required to treat food varies with the specific pathogen and the specific circumstances of the food. It generally takes a higher dose to kill the same number of organisms in frozen food than it does to kill them in refrigerated food. A D-dose is the amount of irradiation that it takes to destroy 90% of the organisms or one decimal log. Thus, a one log kill would reduce a million bacteria to 100,000. Getting rid of more bacteria takes more irradiation as they are small targets and it is not easy to hit each of them. To eliminate 99.999% of the bacteria (a so-called 5-logarithm kill) takes 5 times the irradiation dose needed for a 1 log kill and would reduce a million bacteria to 10. For example, it takes 0.2 kiloGray to reduce *Campylobacter* in meat by one decimal log or 1 kiloGray to reduce it by 5 decimal logs (Table 1).

Irradiation has been approved for use on a broad range of foods for different purposes (Table 2). By an historical quirk, the use of irradiation on food was formally approved as though it were something added to food, rather than a process to which the food is subjected. This means that for meats and poultry, approval is required from both the FDA and USDA. The effect of irradiation on food itself is usually minimal at doses up to 7.5 kGray. Treated food does not become radioactive, and, in general, shelf life is prolonged because organisms that cause spoilage are reduced along with pathogens. Irradiation has been used effectively in meats, poultry, grains, and produce. However, not all foods can be irradiated without changing their quality. Meats with a high fat content may develop off-odors; the whites of eggs may go milky and liquid; and grapefruit gets mushy. Alfalfa seeds do not seem to sprout as well if they are irradiated, and raw oysters may die, which shortens their shelf life substantially.

Table 1. Doses required to decrease selected pathogens at refrigerator temperatures by one decimal log/90% (D-dose)

| Pathogens | D-dose in kGray* | 5-log reduction dose in kGray |
|---|---|---|
| *Campylobacter* | 0.20 | 1.00 |
| *Toxoplasma* cysts | 0.25 | 1.25 |
| *E. coli* O157 | 0.30 | 1.50 |
| *Listeria* | 0.45 | 2.25 |
| *Salmonella* | 0.70 | 2.80 |
| *Cl. botulinum* spores | 3.60 | 18.00 |

* 1 Gray = 100 rad; 10 kGray = 1 megarad

Nutritional and other chemical changes induced in food by irradiation have been studied extensively. In general, these changes are limited to modest declines in the quality and amount of a few vitamins, particularly thiamine (vitamin B1), that are not likely to change the overall adequacy of dietary intake, and to production of transient free radical oxidants, which react almost immediately in the food and do not persist. Similar oxidants are also produced by cooking,

Table 2. Irradiation approved for foods in the United States

| Year | Food | Dose (kGy) | Purpose |
|---|---|---|---|
| 1963 | Wheat flour | 0.20-0.50 | Control mold |
| 1964 | White potatoes | 0.05-0.15 | Inhibit sprouting |
| 1986 | Pork | 0.30-1.00 | Reduce cases of trichinosis |
| 1986 | Fruits and vegetables | 1.00 | Increase shelf life and control insects |
| 1986 | Herbs and spices | 30.00 | Sterilize |
| 1990 (FDA) | Poultry | 3.00 | Reduce bacterial pathogens |
| 1992 (USDA) | Poultry | 1.50-4.50 | Reduce bacterial pathogens |
| 1997 (FDA) | Fresh meat | 4.50 | Reduce bacterial pathogens |
| 2000 (USDA) | Fresh meat | 4.50 | Reduce bacterial pathogens |

and, in any event, would be hydrolyzed immediately in the stomach if any are present. Other radiolytic products are difficult to detect and are present in only trace amounts. It is important to remember that the processes of cooking, such as grilling or frying, themselves induce profound chemical changes in foods, which we depend on to make them edible and tasty. The safety of consuming irradiated foods has been evaluated in large scale trials in animals, some of which lived for several generations (19). No ill effects were observed, and, in particular, no teratogenic effects were seen in mice, hamsters, rats, or rabbits. Formal feeding trials were also conducted with human volunteers without ill effects, and NASA routinely uses irradiated meats in the diet of astronauts.

### Acceptance of Irradiated Foods

Will the public accept irradiated foods? Surveys conducted recently by the Food Marketing Institute and one conducted at FoodNet sites on the general population have had results similar to those obtained in the studies mentioned above (20,21). About 50% of the population is ready to buy irradiated foods, if asked. Acceptance will be greater if irradiated food is not much more expensive than nonirradiated food. The rate of acceptance can increase from 50% up to 80% to 90% if customers understand that irradiation reduces harmful bacteria in food. Similar results have been observed when test marketing irradiated products. Since 2000, irradiated ground beef has been for sale in many markets, and the medical and public health communities can respond to this with enthusiasm.

### Candidates for Food Irradiation

*E. coli* O157 and other Shiga toxin–producing *E. coli* cause more than 100,000 cases of illness per year (2). This infection is untreatable and can lead to severe complications, including hemolytic uremic syndrome, chronic renal failure, and death (22). Ground beef is the most commonly identified source of infection. Pooling the meat of many thousands of animals into ground beef may increase the rate of contamination. Just a few organisms are sufficient to cause severe illness, and efforts to decrease the contamination of ground beef have probably reduced but not eliminated the risk. Irradiating ground beef would effectively destroy *E. coli*.

*Campylobacter jejuni*, the most common of all bacterial foodborne infections, causes an estimated 2,000,000 cases of illness per year (2), and has been associated with Guillain-Barré syndrome (GBS), an acute neurologic disorder (23). Treatment of a *Campylobacter* infection does not prevent its progression to GBS. Poultry is the most commonly identified source of infection. Cross-contamination during slaughter may lead to nearly universal contamination of poultry meat. It takes only a small number of organisms to cause infection. Current efforts to reduce cross-contamination may be responsible for a decrease in *Campylobacter* infections, but these efforts are not likely to eliminate the risk altogether. Irradiating poultry meat would effectively eliminate *Campylobacter* from that food.

*Salmonella*, whose many serotypes are harbored by mammals, birds, and reptiles, causes an estimated 1,400,000 cases of illness and 16,400 hospitalizations per year (2). Up to 2% of humans develop reactive arthropathy after being infected. Foods of animal origin have been the most commonly identified sources, including meat, poultry, eggs, and raw milk (24). Improvements in the safety of egg production and handling have been associated with a recent substantial reduction in the incidence of one common egg-associated serotype, *Salmonella* Enteritidis. Further progress is possible with increased use of eggs pasteurized in their shells, which reached the market in 2000. Improvements in meat and poultry slaughter practices under HACCP may have also had an impact, but they have not eliminated the risk of salmonellosis from raw meat. Irradiating meat and poultry would eliminate *Salmonella* from those foods.

*Listeria monocytogenes* is an opportunistic pathogen that causes an estimated 2,600 cases per year of severe invasive illness (2). This infection affects those who have compromised or undeveloped immune systems, particularly the elderly, the immunocompromised, and pregnant women (25). Approximately 25% of infections lead to death of the immunocompromised patient or loss of the fetus. The number of organisms sufficient to cause infection has not been clearly established. In a healthy host, exposure to extremely high numbers of *Listeria* can result in nothing more than febrile gastroenteritis; in a high-risk individual, a low amount may be sufficient to cause severe infection. The most frequently identified sources are ready-to-eat processed meats and soft cheeses made from unpasteurized milk. Ready-to-eat meats, such as hot dogs, have already been subjected to a pathogen-killing step when the meat is cooked at the factory, so contamination is typically the result of in-plant contamination after that step. Improved sanitation in many plants has reduced the incidence of infection by half since 1986, but the risk persists, as illustrated by a large hot dog–associated outbreak that occurred in 1999 (26). Additional heat treatment or irradiation of meat after it is packaged would eliminate *Listeria* that might be present at that point.

*Toxoplasma gondii* is the most common of all parasitic foodborne infections. As with *Listeria monocytogenes*, the consequences of infection with *T. gondii* are most evident in an immunocompromised person or a pregnant woman (27). Toxoplasmosis causes an estimated 400 to 4,000 cases of congenital disease each year, including hydrocephalus, mental retardation, blindness, and sometimes even death, as well as more than 200,000 noncongenital illnesses, leading to approximately 750 deaths per year, 375 of which may be the consequence of foodborne infections. Consumption of or contact with undercooked meat, especially pork, is an important source of infection, as is contact with feces of an infected cat. Up to 3% of market pigs show serologic evidence of infections or have *Toxoplasma* cysts. Irradiation would inactivate parasites in meat.

### Potential Health Benefits of Irradiating Meat and Poultry

We can roughly estimate the potential benefit of irradiating meat and poultry with a simple calculation. Let us assume that 50% of poultry, ground beef, pork, and processed meats is irradiated. Let us also assume that these foods are the source of 50% of foodborne *E. coli* O157, *Campylobacter*, *Salmonella*, *Listeria,* and *Toxoplasma* infections. The potential benefit of the irradiation would be a 25% reduction in the morbidity and mortality rate caused by these infections (Table 3). This estimated net benefit is substantial, as the measure could prevent nearly 900,000 cases of infection, 8,500 hospitalizations, over 6,000 catastrophic illnesses, and 350 deaths each year. With this estimate we assume that heavily contaminated meat is just as likely to be treated with irradiation as meat which is less contaminated. This estimate does not include the impact on other known pathogens contained in these foods, such as *Yersinia enterocolitica,* or those yet to be identified. This estimate also does not account for the benefits of using irradiation to treat other foods, such as fresh produce that can also be a source of infection.

### Potential Public Concerns about Irradiation

Just as in the early days of milk pasteurization and retort canning, several concerns about this technology have been expressed. Some may ask whether irradiated food is safe to eat. The safety of irradiated food has been studied for four decades, making it the most intensively assessed of any food safety process. Extensive nutritional assessments, toxicity studies, and feeding trials have not indicated a risk, and the process has been approved by many regulatory agencies around the world (28). The nutritional changes produced by irradiation are fewer than those produced by canning or pasteurization.

Others may question the safety of the technology. Irradiation is also used to sterilize products such as surgical implants and instruments; this is a well-established procedure that has been practiced for many years. With regulatory oversight by the Nuclear Regulatory Commission and the Department of Transportation, these procedures are models for how to safely use radioactive sources. Electron beam facilities, which do not have radioactive sources, at this point require less regulatory oversight. Others may wonder if “radiophobia” will prevent the acceptance of irradiation. Actually, most of the American public is prepared to accept irradiation when the benefit of pathogen elimination is clear. For example, it makes sense to use irradiation to sterilize surgical supplies. The population has shown that it is also generally willing to accept X-rays and microwaves as essential to medical diagnosis and convenient cooking, respectively. In the future, a logo indicating that a food product has been irradiated will make it easy for consumers to identify food that has been treated.

Some people may be concerned that gamma ray sources cobalt 60 and cesium 137 are produced in nuclear power reactors. However, this is true of many other radionuclides used routinely in industry, science, and medicine as tracers and treatment agents. Even if nuclear energy is no longer used for commercial power generation in the future, these radionuclides will still be produced in smaller scale reactors. Electron beams and X-rays, of course, do not use radioactive sources and have no link to nuclear energy.

Finally, some people may object to the use of irradiation because it might allow standards to slip in the food industry if irradiation is substituted for other efforts to sanitize the food supply. Actually, combining irradiation with increased sanitation is advantageous because less contamination means lower doses of irradiation would be needed, decreasing the chance of changes in taste or smell of a product. This concern may not be fully resolved until the food industry demonstrates that irradiation is only used in concert with other methods that maintain food sanitation.

Many concerns about irradiation harken back to earlier objections to pasteurization and retort canning. Progressive development in processes and regulations of both technologies ultimately brought about a high measure of safety. The debate between those advocating improved sanitation and those advocating a definitive pathogen reduction technology was finally resolved when both strategies were combined. Instituting pretreatment standards and meat grading would ensure that meat would be clean enough to irradiate. Both milk pasteurization and retort canning became codified with a defined log kill against specific organisms, so that treatment in one place was comparable to treatment in another. Similarly, as the food irradiation industry becomes organized, the process should be defined so that the word “irradiated” will have a standard meaning, thereby ensuring uniform applications. Finally, both pasteurization and retort canning are used to treat food just before or in the final packaging step, at a point when the opportunity for recontamination of the food is minimal. Irradiating food in the same manner will increase confidence that it is not contaminated.

The Centers for Disease Control and Prevention, along with the World Health Organization and many other health organizations, welcomes the use of food irradiation as an important technology that can protect the public against foodborne diseases (28-30). Like pasteurization and retort canning, irradiation is a safe and effective food processing step. Preventing foodborne diseases requires a “farm-to-table” strategy with multiple control steps used along the way. For some foods, this includes a measure that eliminates pathogens definitively. Defined standards and norms for the process of irradiation could enhance general acceptance of this technology, and it would benefit the food industry to begin developing them. Irradiation procedures can be monitored and regulated as are procedures for pasteurization and medical sterilization. The potential benefit of irradiating meat and poultry alone is substantial; it could prevent hundreds of thousands of foodborne illnesses, thousands of hospitalizations, and hundreds of deaths each year. Using these promising technologies is critical to meeting national goals for foodborne disease prevention by 2010.

Table 3. Potential number of health problems prevented annually if 50% of meat and poultry is irradiated

| Pathogen | Cases | Hospitalizations | Major complications | Deaths |
|---|---|---|---|---|
| *E. coli* O157:H7 and other STEC | 23,000 | 700 | At least 250 cases of hemolytic uremic syndrome | 20 |
| *Campylobacter* | 500,000 | 2,600 | 250 cases of GBS | 25 |
| *Salmonella* | 330,000 | 4,000 | 6,000 cases of reactive arthropathy | 140 |
| *Listeria* | 625 | 575 | 60 miscarriages | 125 |
| *Toxoplasma* | 28,000 | 625 | 100-1,000 cases of congenital toxoplasmosis | 94 |
| Total | 881,625 | 8,500 | 6,660 catastrophic illnesses | 352 |

Dr. Tauxe is a medical epidemiologist and chief of the Foodborne and Diarrheal Diseases Branch at the Centers for Disease Control and Prevention.

### References

1. Tauxe RV. Emerging foodborne diseases: an evolving public health challenge. Emerg Infect Dis 1997;3:425-34.
2. Mead PS, Slutsker L, Dietz V, McCaig LF, Bresee JS, Shapiro C, et al. Food-related illness and death in the United States. Emerg Infect Dis 1999;5:607-25.
3. Buzby JC, Roberts T, Lin C-TJ, MacDonald JM. Bacterial foodborne disease: medical costs and productivity losses. Agricultural Economic Report No. 741. Washington: Economic Research Service, U.S. Department of Agriculture, 1996.
4. Committee to Ensure Safe Food from Production to Consumption. Ensuring safe food: from production to consumption. Washington: National Academy Press, 1998.
5. Food safety from farm to table: a national food-safety initiative. Report to the president. Washington: EPA/HHS/USDA, May 1997.
6. Centers for Disease Control and Prevention. Preliminary FoodNet data on the incidence of foodborne illnesses—selected sites, United States, 2000. MMWR Morb Mortal Wkly Rep 2001;50:241-6.
7. Healthy people 2010 objectives: draft for public comment. Office of Public Health and Science. Washington: U.S. Department of Health and Human Services, September 15, 1998.
8. Armstrong C. Botulism from eating canned ripe olives. Public Health Rep 34. 1919;2877-905.
9. Botulism. Protective measures and cautions from the U.S. Bureau of Chemistry, Department of Agriculture. Public Health Rep 1920;35:327-30.
10. Esty JR, Meyer KF. The heat resistance of spores of *B. botulinus* and allied anaerobes, XI. J Infect Dis 1992;31:650-63.
11. McNary-Mapes amendment to the U.S. Food and Drugs Act, fifth paragraph, Section 8, enacted July 8, 1930. Cited in Shrader JH. Food control: its public health aspects. New York: Wiley; 1939. p. 443.
12. Thermally-processed low acid foods packaged in hermetically sealed containers. Fed Reg 2398, January 24, 1973, part 113.
13. Rosenau MJ. Diseases spread by milk. In: Preventive medicine and hygiene. 5th ed. New York: Appleton; 1928. p. 718-26.
14. Potter ME, Kaufmann AF, Blake PA, Feldman RA. Unpasteurized milk: the hazards of a health fetish. JAMA 1984;252:2048-54.
15. U.S. Public Health Service. Milk investigations: preparation of a standard milk-control code. Annual report of the Surgeon General of the Public Health Service of the United States, for the fiscal year 1928. Washington: U.S. Government Printing Office; 1928. p. 53.
16. Headrick ML, Korangy S, Bean NH, Angulo FJ, Altekruse SF, Potter ME, et al. The epidemiology of raw milk-associated foodborne disease outbreaks reported in the United States, 1973 through 1992. Am J Public Health 1998;88:1219-21.
17. Centers for Disease Control and Prevention. Botulism in the United States, 1899-1996. Handbook for epidemiologists, clinicians and laboratory workers, Atlanta: The Centers; 1998.
18. Ackers M-L, Schoenfeld S, Markman J, DeWitt W, Cameron DN, Griffin PM, et al. An outbreak of *Yersinia enterocolitica* O:8 infections associated with pasteurized milk. J Infect Dis 2000;181:1834-7.
19. Josephson ES. An historical review of food irradiation. J Food Safety 1983;5:161.
20. Food Marketing Institute. Consumers' views on food irradiation. Washington: Food Marketing Institute and Grocery Manufacturers of America, 1998.
21. Frenzen P, Buzby J, Majchrowicz A, DeBess E, Hechemy K, Kassenborg H, et al. Consumer acceptance of irradiated meat and poultry in the United States. Program and abstracts book, International Conference on Emerging Infectious Diseases 2000, Atlanta, Georgia; July 16-19, 2000, Abstract, p. 147-8.
22. Griffin PM. Epidemiology of Shiga toxin-producing *Escherichia coli* infections in humans in the United States. Kaper JB, O'Brien A, editors. In *Escherichia coli* O157:H7 and other Shiga toxin-producing *E. coli strains*. Washington: American Society for Microbiology; 1998. p. 15-22.
23. Friedman CR, Neimann J, Wegener HC, Tauxe RV. Epidemiology of *Campylobacter jejuni* infections in the United States and other industrialized nations. In Campylobacter. 2nd ed. Nachamkin I, Blaser MJ, editors. Washington: American Society for Microbiology; 2000. p. 121-38.
24. Tauxe RV, Pavia AT. Salmonellosis: nontyphoidal. In Bacterial infections of humans, epidemiology and control. 3rd ed. Evans AS, Brachman PS, editors. New York: Plenum Medical Book Co.; 1998. p. 223-42.
25. Slutsker L, Schuchat A. Listeriosis in humans. In Listeria, listeriosis and food safety, 2nd ed. Ryser ET, Marth EH, editors. New York: Marcel Dekker; 1999. p. 75-95.
26. Centers for Disease Control and Prevention. Multistate outbreak of listeriosis—United States, 1998. MMWR Morb Mortal Wkly Rep 1998;47:1085-6.
27. Lopez A, Dietz VJ, Wilson M, Navin TR, Jones JL. Preventing congenital toxoplasmosis. MMWR Morb Mortal Wkly Rep 2000;49:RR-02,57-75.
28. Steele JH. Food irradiation: a public health opportunity. Int J Infect Dis 2000;4:62-6.
29. Osterholm MT, Potter ME. Irradiation pasteurization of solid foods: taking food safety to the next level. Emerg Infect Dis 1997;3:575-7.
30. World Health Organization. Safety and nutritional adequacy of irradiated food. Geneva: The Organization; 1984.
